# Theory of planned behaviour-based interventions in chronic diseases among low health-literacy population: protocol for a systematic review

**DOI:** 10.1186/s13643-022-02006-2

**Published:** 2022-06-21

**Authors:** Biswajit Paul, Richard Kirubakaran, Rita Isaac, Marshall Dozier, Liz Grant, David Weller, RESPIRE collaboration

**Affiliations:** 1 Christian Medical College Vellore, Vellore, India; 2 University of Edinburgh, Edinburgh, UK

**Keywords:** Protocol, Chronic diseases, Theory of planned behaviour/TPB, Behaviour change, Low- and middle-income countries/LMICs

## Abstract

**Background:**

Health behaviour can change outcomes in both healthy and unhealthy populations and are particularly useful in promoting compliance to treatment and maintaining fidelity to care seeking and follow-up options in chronic diseases. Interventions to change health behaviour based on psychological theory are more often successful than those without any underlying theory. The theory of planned behaviour (TPB) is one such psychological theory which had been found to predict human behaviour with respect to disease prevention and when applied to interventions can change the outcomes of diseases. Most of the research evidence of TPB-based interventions have been from developed world. Evidence is required whether TPB-based interventions can be applied and works in low-resource, low health-literacy settings of low- and middle-income countries (LMICs).

**Methods:**

The protocol has been developed as per PRISMA-P guidelines and incorporates PICO (*p*opulation, *i*ntervention, *c*omparison, *o*utcomes) framework for describing the methodology. Population above 18 years of age and having any chronic disease (as defined for this systematic review) will be selected, while any health or educational intervention based on constructs of TPB will be included. Comparison will be with non-TPB-based interventions or treatment as usual without any intervention, and the primary outcome will be the behaviour change effected by the TPB-based intervention. Intervention studies will be considered, and relevant databases like MEDLINE, Embase, Cochrane Library and ProQuest will be explored. Data extraction will done in a standardised form, and risk-of-bias assessment will be done using the Cochrane Collaboration’s tools for such assessment. Narrative synthesis of the selected studies will be done to draw the conclusions, and meta-analysis will be done to calculate the effect estimates with I-squared statistics to describe the heterogeneity.

**Discussion:**

This systematic review will provide new evidence on fidelity and effectiveness of the TPB-based interventions among chronic disease patients from low health literacy, resource-poor background. It will inform of how to plan and use such interventions to change health behaviour in chronic disease patients, particularly in LMIC settings.

**Systematic review registration:**

PROSPERO CRD42018104890.

**Supplementary Information:**

The online version contains supplementary material available at 10.1186/s13643-022-02006-2.

## Background

Chronic diseases including noncommunicable diseases affect all age groups, countries and regions; however, they are associated mainly with older age groups and cause most of the premature deaths (~ 85%) in low- and middle-income countries [1]. Chronic diseases have been defined variedly by different organisations and include multiple conditions which may vary between agencies or researchers [2–5]. In this systematic review, we have used the definition of chronic diseases used by World Health Organization [3] and have included major chronic diseases. Chronic diseases are usually of longer duration; require prolonged treatment, repeated hospital visits and follow-up; and involve specific health behaviours for prevention or control. Health behaviour is the underpinning component which can modify or influence many of the risk factors, lead to better management of chronic diseases and improve compliance to treatment [6].

Health behaviour refers to any behaviour that impacts on people’s physical and mental health and quality of life [7]. It is defined by Gochman (1997) as “ personal attributes such as beliefs, expectations, motives, values, perceptions, and other cognitive elements; personality characteristics, including affective and emotional states and traits; and overt behaviour patterns, actions, and habits that relate to health maintenance, to health restoration, and to health improvement” [8]. Health behaviour influences health outcomes in both healthy and unhealthy populations, whereas in healthy population, they are primarily important in prevention of diseases and promotion of health; in population with diseases, they can influence quality of life. Three types of behaviour are related to population health: first, behaviours which contribute to the prevention of disease; second, behaviours which involve care seeking and adherence to treatment; and, third, behaviours that relate to the delivery of healthcare [7]. Behaviours which involve care seeking and adherence to treatment have key influences on the health of population with chronic diseases.

Adopting appropriate health behaviours is critical to avoid risk factors, seek treatment and continue follow-up in chronic diseases. Theory-based interventions targeting health behaviours have been shown to be more effective than those without [9, 10] and provides a useful framework for identifying the key modifiable determinants of health behaviour [11].

One of the key determinants of health behaviour is health literacy. It is defined as “the degree to which individuals can obtain, process, and understand the basic health information and services they need to make appropriate health decisions” [12]. Health literacy have been found to be a predictor for adopting preventive health behaviours like accepting screening tests or adopting physical activity [13]. Studies have shown that health literacy can influence health services and interventions to improve health behaviour in chronic diseases and promote self-care behaviour and desirable health outcomes [14–16]. Highlighting the health impact of low health literacy, a 2004 systematic evidence review [17] and its update in 2011 [12] found a relationship between low health-literacy and poor health outcomes. According to the US Department of Education survey done in 2003, approximately 80 million adults in the USA have limited health literacy, with groups like the elderly, minorities, individuals who have not completed high school, adults who spoke a language other than English before starting school and people living in poverty having a higher prevalence [18]. Health literacy in low- or middle-income countries (LMICs) is lower than that measured in the USA and other high-income countries (HICs), because by definition, general income and education of people in LMIC will be lower as a whole as well [19–21].

It is important to look into theory-based interventions, particularly theory of planned behaviour (TPB)-based interventions used to change health behaviour in people with chronic disease in the LMIC settings (with presumably lower-health literacy) in the context of the ever-increasing global burden of chronic diseases. The TPB focuses on theoretical constructs concerned with individual motivational factors and capability as determinants of the likelihood of performing a specific behaviour. TPB assumes that the best predictor of human behaviour is behavioural intention which in turn is determined by attitude towards the behaviour, social normative perceptions regarding it and perceived control over performance of the behaviour. Interventions based on TPB have been found to be effective in changing health behaviours [22]. TPB has been found to predict if an individual engages in a wide variety of different health behaviours including exercise, undergoing a health check-up and being screened for breast and colorectal cancers [23, 24]. TPB-based interventions have improved outcomes in diseases like obesity and schizophrenia and health behaviours like fruit and vegetable intake and exercise patterns [25, 26]. Systematic review on TPB-based interventions in LMIC settings is essential to understand how these interventions work in low literacy, poor and/or rural populations, how effective they are in such settings and do they require different kinds of implementation; currently, the available information is from high-income countries which may not be suitable to develop a health/educational intervention for LMICs.

## Methods

### Aim

The aim of this review is to evaluate the effect of TPB-based interventions on changing health behaviour among population with chronic diseases in low health-literacy settings. This study also aims at finding out which types of interventions were used, the time frame of such interventions, the modes of delivery and the settings in which these interventions were delivered. This protocol has been developed in accordance with the Preferred Reporting Items for Systematic review and Meta-Analysis Protocols (PRISMA-P) guidance [27] and draws on the *Cochrane Handbook for Systematic Reviews of Interventions* guidelines for developing a protocol [28].

### Study types and participants

The following study types will be considered for inclusion: interventional studies with a control arm including randomised controlled trials, quasi-experimental studies, community-based cluster randomised trials and controlled before and after studies. Case–control studies, cohort studies, reviews, case reports, case series and animal studies will be excluded. Animal studies, studies on health behaviour change which do not mention TPB or other psychological theories and studies undertaken on healthy individuals with a purely health promotion focus will be not considered in this review.

Adult participants 18 years of age or more with chronic disease(s)) [29] will be part of this review; chronic diseases including cardiovascular diseases, cancers, chronic respiratory diseases, diabetes, hypertension, obesity, Alzheimer’s disease, osteoarthritis, urinary incontinence and HIV/AIDS will be considered for this systematic review. Healthy population and pregnant women will be excluded.

### Intervention and control groups

Any educational or health intervention is used on individuals or groups that documented the use of the constructs of TPB, i.e. attitude towards the behaviour, subjective norms and perceived behavioural control for changing health behaviour. From a scoping search of literature, the following terms for identifying TPB will be used: behavioural beliefs, normative beliefs, control beliefs, motivation to comply and perceived power and behavioural intention, besides attitude towards the behaviour, subjective norms and perceived behavioural control.

The control groups will have either (1) any health or educational intervention not based on any psychological theory, (2) health education based on psychological theory other than TPB or (3) treatment as usual without any education. The same group may also be used as control before the TPB-based intervention was delivered to the group and the outcomes evaluated.

### Outcomes

The primary outcome will be change in health behaviour which will include preventive behaviours, adherence to treatment and care seeking. Adherence has been defined by the World Health Organization (WHO) as “the extent to which a person’s behaviour – taking medication, following a diet, and/or executing lifestyle changes, corresponds with agreed recommendations from a health care provider” [30].

Secondary outcomes will include the constructs of TPB which were important influences to health behaviour change and moderators of these effects, including type of intervention, the time-frame of such interventions, fidelity of the intervention, i.e. proportion of people completing the intervention, the types of health literacy settings, the mode of delivery, the type of providers and satisfaction among patients to the intervention.

### Study identification and selection

The following databases will be searched for relevant studies: MEDLINE, Embase, Cochrane Library, PsychINFO, Web of Science, Scopus, CINAHL, ProQuest databases (ProQuest Sociology and ProQuest Social Sciences), Global Index Medicus, Bibliography of Asian Studies and IndMED. We will also search the grey literature through Open Grey and the Grey Literature Report. Search strategies will be developed for all the databases.

The databases will be searched for relevant studies, and all such records will be exported to the EndNote Library for screening, deduplication and overall management of the records. All the studies will be screened by two independent reviewers, and any disagreement will be resolved by discussion and if necessary will be resolved by arbitration by a third reviewer. A similar process will be followed for full screening of full-text studies. Multiple publications of the same study utilising the same data set will be taken as one study. In case of missing data, efforts will be made to contact the authors to request the data. If the full-text article for a particular study title or abstract is not freely available through our library resources, external request through an interlibrary loan will be made; if this is not successful, the authors will be approached to provide the full-text article. Studies arriving after the cut-off date will be not included, but their titles will be mentioned along with the reasons for noninclusion.

### Data extraction, management and ‘risk-of-bias’ assessment

A data extraction form will be developed and standardised; it will be piloted and revised before the start of the review. The data extraction form will be adapted from the Cochrane Collaboration data collection form of randomised controlled trials (RCTs) and non-randomised studies (NRS) [31]. Data extraction will be performed by two independent reviewers, and any discrepancies will be resolved by discussion; if still disagreement persists, it will be arbitrated by a third reviewer.

Risk of bias for all potential studies will be evaluated using the Cochrane Collaboration’s tools for assessing the risk of bias. For randomised controlled trials (RCTs), revised Cochrane risk-of-bias tool for randomised trials (RoB 2) will be used [31], while for non-randomised studies like quasi-experimental studies and controlled before and after studies, the risk of bias in non-randomised studies of interventions (ROBINS-I) tool will be used [32]. As per the guidelines in the *Cochrane Handbook of Systematic Reviews of Interventions*, the studies will be assessed as per standard criteria and will be labelled as ‘low’, ‘unclear’ or ‘high’ risk of bias.

### Analysis

All the characteristics of included studies will be presented in a tabular form with the description of study design, type of disease, type of intervention used, no. of groups involved, outcomes and methods of assessment, and risk of bias in each study. The fidelity of the studies will also be assessed, i.e. the number of people completing the intervention and reasons for noncompliance. We will do a narrative synthesis of the studies to draw the conclusions. Whether or not we will do a pooled quantitative estimation (meta-analysis) will depend on the type and heterogeneity of the studies. We will follow the guidance provided in the handbook of Cochrane systematic review to evaluate the heterogeneity. As we expect a high level of heterogeneity, we will apply random effect model. If we do meta-analysis, I-squared statistics will be used to report the heterogeneity of results. We intend to explore the reason for heterogeneity with the following subgroup analysis — low health literacy, types of intervention and modes of delivery; this will help explore heterogeneity within groups, which may alter the results of the intervention. We want to explore the difference in health behaviour because of the intervention due to literacy rates among populations and due to type of interventions like randomised controlled trials, cluster randomised trials and controlled before and after studies. The third area where we think sub-group analysis will be useful is the mode of delivery of intervention, e.g. online versus face-to-face and hybrid intervention delivery which might be useful to explore during this COVID-19 pandemic era. We will compare the effect estimates in different subgroups by considering the meta-analysis results from each subgroup separately.

We intend to do a sensitivity analysis by excluding high risk-of-bias studies to evaluate the robustness of the overall pooled estimate.

### Grading of overall strength of evidence

We will attempt to apply the Grading of Recommendations Assessment, Development and Evaluation (GRADE) approach [31] for creating a summary of findings table. The GRADEpro Guideline Development Tool (GDT), an online tool, will be used for either importing the data from RevMan or manually adding numerical data into the software for each outcome. The GRADE approach has five domains: risk of bias, inconsistency, indirectness, imprecision and publication bias to evaluate the certainty of evidence for randomised studies. The four levels of evidence of GRADE will be applied, i.e. very low, low, moderate and high.

### Registration and reporting

The study is registered with the University of York Centre for Reviews and Dissemination international prospective register of systematic reviews (PROSPERO) with registration no. CRD42018104890. The review will be reported in accordance with the PRISMA guidelines for reporting of systematic reviews. Any amendments to the protocol made during the process of the systematic review will be reported in the final report with rationale for such modifications.

### The review team

The search for databases and retrieval of studies, screening, data extraction and quality assessment will be performed independently by BP and RK. BP will work under the guidance of MD. RK is an experienced reviewer working with Cochrane Collaboration — South Asia and well experienced in conducting systematic reviews. MD, DW or RI will arbitrate any disagreements in the review process and will provide field expertise in synthesising the data. DW and RI are experienced researchers; DW and MD have a substantial experience in undertaking systematic reviews.

## Discussion

It is known that TPB is effective in changing behaviours, and there is evidence on ways the different constructs of TPB explain these changes. However, there is little specific information on the change in health behaviour brought about by TPB-based interventions in chronic diseases and their applicability in different settings — in particular, LMICs. This review will add to the evidence base of understanding and applying the TPB-based interventions in changing health behaviour in chronic diseases in low health literacy settings which will inform prevention and treatment approaches as well as intervention development for behaviour change in such settings.

### Existing reviews in this field

Three other similar reviews have been undertaken. Wendy Hardeman et al. [33] examined interventions based on TPB to change health behaviour; however, it focussed on behaviour change on any population where TPB has been applied without any mention of chronic diseases. This review, conducted in 2001, also indicated that TPB was mainly used to measure process and outcome variables and to predict intention and behaviour and less commonly to develop the intervention.

A second, more recent review, by Steinmetz et al. [26], incorporated a three-level meta-analysis to establish that interventions based on TPB were effective in changing behaviour; there was a mean effect size of 0.50 and effect sizes ranging from 0.14 to 0.68 for changes in antecedent variables (behavioural, normative, and control beliefs, attitude, subjective norm, perceived behavioural control and intention). This review examined behaviour change across all behavioural domains and types of conditions were not specified.

Thirdly, Antonia Rich et al. [34] examined, in 2015, the role of TPB in predicting adherence in people with a chronic condition. The review suggested that TPB makes a useful contribution to our understanding of adherence in chronic illness; it measured the types of adherence behaviours, adherence measures and the effects of the TPB constructs on adherence behaviour. However, it did not specify interventions were based solely on TPB but considered any type of study referencing TPB and using any of the constructs of TPB. Furthermore, it did not examine the settings in which the interventions were delivered or had any reference to health literacy and excluded studies with populations considered to be at risk of chronic disease (e.g. sedentary adults).

The present review looks to evaluate the role of TPB-based interventions in patients with chronic diseases and the health behaviour change that may occur in such cases, particularly in low health-literacy populations of low- and middle-income countries (LMICs). It also seeks to describe the type of change, the settings and moderators for such change and the types of interventions effecting such change.

## Conclusion

The burden of death and disability due to chronic diseases is increasing throughout the world with majority of the burden being borne by LMICs. Many of these patients suffer lifelong from these conditions, and changing health behaviour can improve the quality of life of such individuals, typically by applying theory of planned behaviour-based interventions. Evidence is needed about the applicability of TPB-based interventions for behaviour change in chronic diseases in low health-literacy settings — and we need to understand the different moderators influencing such change. This review will help in gathering evidence in these under-reviewed areas and help researchers and policymakers to plan such intervention programmes for both prevention and treatment.

## Supplementary Information

**Additional file 1.** Search Strategy MEDLINE. Describes the step wise process for screening relevant articles from MEDLINE for the systematic review. This strategy is also adapted for searching other databases and is reproduced here as an example.

**Additional file 2.** Data extraction form and risk of bias tools, ROBBINS-I and RoB 2. This describes the data extraction form which is being modified and adapted from the Cochrane Collaboration Data collection for intervention review and the Cochrane Collaboration Risk of Bias tool for quality assessment.

**Additional file 3.** PRISMA-P checklist. This is a populated PRISMA-P checklist which captures the basic requirements of a systematic review protocol and mentions by page numbers, each item of the checklist in the manuscript, which gives a fair idea to the readers about the process.

## Data Availability

Data sharing is not applicable to this article as no datasets were generated or analysed during the current study.

## Abbreviations

GDT: Guideline Development Tool
GRADE: Grading of Recommendations Assessment, Development and Evaluation
HIC: High-income country
LMIC: Low- and middle-income country
PRISMA-P: Preferred Reporting Items for Systematic review and Meta-Analysis Protocols
PROSPERO: International prospective register of systematic reviews
RCT: Randomised control trial
TPB: Theory of planned behaviour
WHO: World Health Organization
