# Supplementary material for: Theory of planned behaviour-based interventions in chronic diseases among low health-literacy population: protocol for a systematic review
Source: Syst Rev. 2022 Jun 21;11:127. doi: 10.1186/s13643-022-02006-2 (PMC9210335; doi:10.1186/s13643-022-02006-2)
Supplement: Supplementary file 2 — Additional file 2. Data extraction form and risk of bias tools, ROBBINS-I and RoB 2. This describes the data extraction form which is being modified and adapted from the Cochrane Collaboration Data collection for intervention review and the Cochrane Collaboration Risk of Bias tool for quality assessment. [file 13643_2022_2006_MOESM2_ESM.pdf]

Additional File 2
Data extraction form and risk of bias tools

Data extraction form

Identification

Review title or ID	
Study ID *(surname of first author and year first full report of study was published e.g. Smith 2001)*	
Report ID	
Report ID of other reports of this study including errata or retractions	
Notes	

General Information

Date form completed *(dd/mm/yyyy)*	
Name/ID of person extracting data	
Reference citation	
Study author contact details	
Publication type *(e.g. full report, abstract, letter)*	
Notes:	

Study Eligibility

Study Characteristics	Eligibility criteria *(Insert inclusion criteria for each characteristic as defined in the Protocol)*	Eligibility criteria met? Yes	No	Unclear	Location in text or source *(pg & ¶/fig/table/other)*
Type of study	Randomised Controlled Trial	☐	☐	☐	
	Quasi-randomised Controlled Trial	☐	☐	☐	
	Cluster Randomised Trial	☐	☐	☐	
	Controlled Before and After Study<br>Contemporaneous data collection<br>Comparable control sites	☐	☐	☐	

	At least 2 x intervention and 2 x control clusters		
	Interrupted Time Series<br>At least 3 time points before and 3 after the intervention<br>Clearly defined intervention point	☐ ☐ ☐	
	Other design (specify):	☐ ☐ ☐	
Types of intervention	TPB based intervention	☐ ☐ ☐	
	At least 2 of the 3 constructs of TPB used by the intervention	☐ ☐ ☐	
	Intervention using multiple psychological theories with clearly measurable TPB constructs	☐ ☐ ☐	
Types of comparison / control	Health education not based on any psychological theory	☐ ☐ ☐	
	Health education based on psychological theory other than TPB	☐ ☐ ☐	
	Treatment as usual without any structured health education	☐ ☐ ☐	
Participants	Adults above 18 years of age, any gender and not Caucasian<br>Should have any chronic disease	☐ ☐ ☐	
Study setting	The geographical location of the study should be a LMIC	☐ ☐ ☐	
Types of outcome measures	Knowledge	☐ ☐ ☐	
	Attitude, subjective norms, perceived behavioural control	☐ ☐ ☐	
	Health behaviour *(e.g. exercise, medication use, smoking cessation, inhaler use)*	☐ ☐ ☐	
INCLUDE ☐		EXCLUDE ☐	

Reason for exclusion	
Notes:	

DO NOT PROCEED IF STUDY EXCLUDED FROM REVIEW

Characteristics of included studies

Methods

	Descriptions as stated in report/paper		Location in text or source *(pg & ¶/fig/table/other)*
**Aim of study** *(e.g. efficacy, equivalence, pragmatic)*			
**Design** *(e.g. parallel, crossover, non-RCT)*			
**Unit of allocation** *(by individuals, cluster/ groups or body parts)*			
**Start date**			
**End date**			
**Duration of participation** *(from recruitment to last follow-up)*			
**Ethical approval needed/ obtained for study**	☐ Yes ☐ No ☐ Unclear		
**Notes:**			

Participants

	Description<br>*Include comparative information for each intervention or comparison group if available*		Location in text or source *(pg & ¶/fig/table/other)*
Population description *(from which study participants are drawn)*			
Setting *(including location and social context)*			
Inclusion criteria			
Exclusion criteria			
Method of recruitment of participants *(e.g. phone, mail, clinic patients)*			
Informed consent obtained	☐ Yes ☐ No ☐ Unclear		
Total no. randomised *(or total pop. at start of study for NRCTs)*			
Clusters *(if applicable, no., type, no. people per cluster)*			
Baseline imbalances			
Withdrawals and exclusions *(if not provided below by outcome)*			
Age			
Sex			

Race/Ethnicity		
Severity of illness		
Co-morbidities		
Other relevant socio-demographics		
Subgroups measure		
Subgroups reported		
Notes:		

Intervention groups

Copy and paste table for each intervention and comparison group

Intervention Group 1

	Description as stated in report/paper	Location in text or source *(pg & ¶/fig/table/other)*
Group name		
No. randomised to group *(specify whether no. people or clusters)*		
Theoretical basis *(include key references)*		
Description *(include sufficient detail for replication, e.g. content, dose, components)*		
Duration of treatment period		
Timing *(e.g. frequency, duration of each episode)*		
Delivery *(e.g. mechanism, medium, intensity, fidelity)*		

Providers *(e.g. no., profession, training, ethnicity etc. if relevant)*		
Co-interventions		
Economic information *(i.e. intervention cost, changes in other costs as result of intervention)*		
Resource requirements *(e.g. staff numbers, cold chain, equipment)*		
Integrity of delivery		
Compliance		
Notes:		

Outcomes

Copy and paste table for each outcome.

Outcome 1

	Description as stated in report/paper	Location in text or source *(pg & ¶/fig/table/other)*
Outcome name		
Time points measured *(specify whether from start or end of intervention)*		
Time points reported		
Outcome definition *(with diagnostic criteria if relevant)*		
Person measuring/ reporting		
Unit of measurement *(if relevant)*		

Scales: upper and lower limits *(indicate whether high or low score is good)*		
Is outcome/tool validated?	☐ Yes ☐ No ☐ Unclear		
Imputation of missing data *(e.g. assumptions made for ITT analysis)*		
Assumed risk estimate *(e.g. baseline or population risk noted in Background)*		
Power *(e.g. power & sample size calculation, level of power achieved)*		
Notes:		

Other

**Study funding sources** *(including role of funders)*		
**Possible conflicts of interest** *(for study authors)*		
**Notes:**		

Risk of Bias assessment

The Risk Of Bias In Non-randomized Studies – of Interventions (ROBINS-I) assessment tool

ROBINS-I tool (Stage I): At protocol stage

Specify the review question

Participants	
Experimental intervention	
Comparator	
Outcomes	

List the confounding domains relevant to all or most studies

List co-interventions that could be different between intervention groups and that could impact on outcomes

ROBINS-I tool (Stage II): For each study

Specify a target randomized trial specific to the study

Design	Individually randomized / Cluster randomized / Matched (e.g. cross-over)
Participants	
Experimental intervention	
Comparator	

Is your aim for this study…?

☐ to assess the effect of *assignment to* intervention
☐ to assess the effect of *starting and adhering to* intervention

Specify the outcome

Specify which outcome is being assessed for risk of bias (typically from among those earmarked for the Summary of Findings table). Specify whether this is a proposed benefit or harm of intervention.

Specify the numerical result being assessed

In case of multiple alternative analyses being presented, specify the numeric result (e.g. RR = 1.52 (95% CI 0.83 to 2.77) and/or a reference (e.g. to a table, figure or paragraph) that uniquely defines the result being assessed.

Preliminary consideration of confounders

Complete a row for each important confounding domain (i) listed in the review protocol; and (ii) relevant to the setting of this particular study, or which the study authors identified as potentially important.

"Important" confounding domains are those for which, in the context of this study, adjustment is expected to lead to a clinically important change in the estimated effect of the intervention.

"Validity" refers to whether the confounding variable or variables fully measure the domain, while "reliability" refers to the precision of the measurement (more measurement error means less reliability).

**(i) Confounding domains listed in the review protocol**				
Confounding domain	Measured variable(s)	Is there evidence that controlling for this variable was unnecessary?*	Is the confounding domain measured validly and reliably by this variable (or these variables)?	OPTIONAL: Is failure to adjust for this variable (alone) expected to favour the experimental intervention or the comparator?
			Yes / No / No information	Favour experimental / Favour comparator / No information

**(ii) Additional confounding domains relevant to the setting of this particular study, or which the study authors identified as important**

Confounding domain	Measured variable(s)	Is there evidence that controlling for this variable was unnecessary?*	Is the confounding domain measured validly and reliably by this variable (or these variables)?	OPTIONAL: Is failure to adjust for this variable (alone) expected to favour the experimental intervention or the comparator?
			Yes / No / No information	Favour experimental / Favour comparator / No information

* In the context of a particular study, variables can be demonstrated not to be confounders and so not included in the analysis: (a) if they are not predictive of the outcome; (b) if they are not predictive of intervention; or (c) because adjustment makes no or minimal difference to the estimated effect of the primary parameter. Note that “no statistically significant association” is not the same as “not predictive”.

Preliminary consideration of co-interventions

Complete a row for each important co-intervention (i) listed in the review protocol; and (ii) relevant to the setting of this particular study, or which the study authors identified as important.

"Important" co-interventions are those for which, in the context of this study, adjustment is expected to lead to a clinically important change in the estimated effect of the intervention.

**(i) Co-interventions listed in the review protocol**		
Co-intervention	Is there evidence that controlling for this co-intervention was unnecessary (e.g. because it was not administered)?	Is presence of this co-intervention likely to favour outcomes in the experimental intervention or the comparator
		Favour experimental / Favour comparator / No information
		Favour experimental / Favour comparator / No information
		Favour experimental / Favour comparator / No information
		Favour experimental / Favour comparator / No information

**(ii) Additional co-interventions relevant to the setting of this particular study, or which the study authors identified as important**		
Co-intervention	Is there evidence that controlling for this co-intervention was unnecessary (e.g. because it was not administered)?	Is presence of this co-intervention likely to favour outcomes in the experimental intervention or the comparator
		Favour experimental / Favour comparator / No information
		Favour experimental / Favour comparator / No information
		Favour experimental / Favour comparator / No information
		Favour experimental / Favour comparator / No information

Risk of bias assessment

Responses underlined in green are potential markers for low risk of bias, and responses in red are potential markers for a risk of bias. Where questions relate only to sign posts to other questions, no formatting is used.

Signalling questions	Description
**Bias due to confounding**	
1.1 Is there potential for confounding of the effect of intervention in this study?<br>**If** N/PN **to 1.1:** the study can be considered to be at low risk of bias due to confounding and no further signalling questions need be considered	
**If** Y/PY **to 1.1**: determine whether there is a need to assess time-varying confounding:	
1.2. Was the analysis based on splitting participants' follow up time according to intervention received?<br>**If N/PN**, answer questions relating to baseline confounding (1.4 to 1.6)<br>**If Y/PY**, go to question 1.3.	
1.3. Were intervention discontinuations or switches likely to be related to factors that are prognostic for the outcome?<br>**If N/PN**, answer questions relating to baseline confounding (1.4 to 1.6)<br>**If Y/PY**, answer questions relating to both baseline and time-varying confounding (1.7 and 1.8)	

**Questions relating to baseline confounding only**	
1.4. Did the authors use an appropriate analysis method that controlled for all the important confounding domains?	
1.5. **If** Y/PY **to 1.4**: Were confounding domains that were controlled for measured validly and reliably by the variables available in this study?	
1.6. Did the authors control for any post-intervention variables that could have been affected by the intervention?	
**Questions relating to baseline and time-varying confounding**	
1.7. Did the authors use an appropriate analysis method that controlled for all the important confounding domains and for time-varying confounding?	
1.8. **If** Y/PY **to 1.7**: Were confounding domains that were controlled for measured validly and reliably by the variables available in this study?	
**Risk of bias judgement**	
Optional: What is the predicted direction of bias due to confounding?	

**Bias in selection of participants into the study**	
2.1. Was selection of participants into the study (or into the analysis) based on participant characteristics observed after the start of intervention?<br>**If** N/PN **to 2.1:** go to 2.4	

2.2. **If Y/PY to 2.1**: Were the post-intervention variables that influenced selection likely to be associated with intervention?<br>2.3 **If Y/PY to 2.2**: Were the post-intervention variables that influenced selection likely to be influenced by the outcome or a cause of the outcome?	
2.4. Do start of follow-up and start of intervention coincide for most participants?	
2.5. **If Y/PY to 2.2 and 2.3, or N/PN to 2.4**: Were adjustment techniques used that are likely to correct for the presence of selection biases?	
**Risk of bias judgement**	
Optional: What is the predicted direction of bias due to selection of participants into the study?	

**Bias in classification of interventions**	
3.1 Were intervention groups clearly defined?	
3.2 Was the information used to define intervention groups recorded at the start of the intervention?	
3.3 Could classification of intervention status have been affected by knowledge of the outcome or risk of the outcome?	
**Risk of bias judgement**	
Optional: What is the predicted direction of bias due to classification of interventions?	

**Bias due to deviations from intended interventions**	
**If your aim for this study is to assess the effect of assignment to intervention, answer questions 4.1 and 4.2**	
4.1. Were there deviations from the intended intervention beyond what would be expected in usual practice?	
4.2. **If Y/PY to 4.1**: Were these deviations from intended intervention unbalanced between groups *and* likely to have affected the outcome?	
**If your aim for this study is to assess the effect of starting and adhering to intervention, answer questions 4.3 to 4.6**	
4.3. Were important co-interventions balanced across intervention groups?	
4.4. Was the intervention implemented successfully for most participants?	
4.5. Did study participants adhere to the assigned intervention regimen?	
4.6. **If N/PN to 4.3, 4.4 or 4.5**: Was an appropriate analysis used to estimate the effect of starting and adhering to the intervention?	
**Risk of bias judgement**	
Optional: What is the predicted direction of bias due to deviations from the intended interventions?	

**Bias due to missing data**

5.1 Were outcome data available for all, or nearly all, participants?	
5.2 Were participants excluded due to missing data on intervention status?	
5.3 Were participants excluded due to missing data on other variables needed for the analysis?	
5.4 **If PN/N to 5.1, or Y/PY to 5.2 or 5.3**: Are the proportion of participants and reasons for missing data similar across interventions?	
5.5 **If PN/N to 5.1, or Y/PY to 5.2 or 5.3**: Is there evidence that results were robust to the presence of missing data?	
**Risk of bias judgement**	
Optional: What is the predicted direction of bias due to missing data?	

**Bias in measurement of outcomes**	
6.1 Could the outcome measure have been influenced by knowledge of the intervention received?	
6.2 Were outcome assessors aware of the intervention received by study participants?	
6.3 Were the methods of outcome assessment comparable across intervention groups?	
6.4 Were any systematic errors in measurement of the outcome related to intervention received?	
**Risk of bias judgement**	
Optional: What is the predicted direction of bias due to measurement of outcomes?	

**Bias in selection of the reported result**	
Is the reported effect estimate likely to be selected, on the basis of the results, from... 7.1. ... multiple outcome *measurements* within the outcome domain?	
7.2 ... multiple *analyses* of the intervention-outcome relationship?	
7.3 ... different *subgroups*?	
**Risk of bias judgement**	
Optional: What is the predicted direction of bias due to selection of the reported result?	

**Overall bias**	
**Risk of bias judgement**	
Optional: What is the overall predicted direction of bias for this outcome?	

Revised Cochrane risk-of-bias tool for randomized trials (RoB 2)

Responses underlined in green are potential markers for low risk of bias, and responses in red are potential markers for a risk of bias. Where questions relate only to sign posts to other questions, no formatting is used.

Signalling questions	Comments	Response options
**1.1 Was the allocation sequence random?**		Y / PY / PN / N / NI
**1.2 Was the allocation sequence concealed until participants were enrolled and assigned to interventions?**		Y / PY / PN / N / NI
**1.3 Did baseline differences between intervention groups suggest a problem with the randomization process?**		Y / PY / PN / N / NI
**Risk-of-bias judgement**		Low / High / Some concerns
Optional: What is the predicted direction of bias arising from the randomization process?		NA / Favours experimental / Favours comparator / Towards null /Away from null / Unpredictable

Domain 1: Risk of bias arising from the randomization process

Domain 2: Risk of bias due to deviations from the intended interventions (effect of assignment to intervention)

Signalling questions	Comments
**2.1. Were participants aware of their assigned intervention during the trial?**	
**2.2. Were carers and people delivering the interventions aware of participants' assigned intervention during the trial?**	
**2.3. If Y/PY/NI to 2.1 or 2.2: Were there deviations from the intended intervention that arose because of the trial context?**	
**2.4 If Y/PY to 2.3: Were these deviations likely to have affected the outcome?**	
**2.5. If Y/PY/NI to 2.4: Were these deviations from intended intervention balanced between groups?**	
**2.6 Was an appropriate analysis used to estimate the effect of assignment to intervention?**	
**2.7 If N/PN/NI to 2.6: Was there potential for a substantial impact (on the result) of the failure to analyse participants in the group to which they were randomized?**	
**Risk-of-bias judgement**	
Optional: What is the predicted direction of bias due to deviations from intended interventions?	

Domain 2: Risk of bias due to deviations from the intended interventions (effect of adhering to intervention)

Signalling questions	Comments
**2.1. Were participants aware of their assigned intervention during the trial?**	
**2.2. Were carers and people delivering the interventions aware of participants' assigned intervention during the trial?**	
**2.3. [If applicable:] If Y/PY/NI to 2.1 or 2.2: Were important non-protocol interventions balanced across intervention groups?**	
**2.4. [If applicable:] Were there failures in implementing the intervention that could have affected the outcome?**	
**2.5. [If applicable:] Was there non-adherence to the assigned intervention regimen that could have affected participants' outcomes?**	
**2.6. If N/PN/NI to 2.3, or Y/PY/NI to 2.4 or 2.5: Was an appropriate analysis used to estimate the effect of adhering to the intervention?**	
**Risk-of-bias judgement**	
Optional: What is the predicted direction of bias due to deviations from intended interventions?	

Domain 3: Missing outcome data

Signalling questions	Comments
**3.1 Were data for this outcome available for all, or nearly all, participants randomized?**	
**3.2 If N/PN/NI to 3.1: Is there evidence that the result was not biased by missing outcome data?**	
**3.3 If N/PN to 3.2: Could missingness in the outcome depend on its true value?**	
**3.4 If Y/PY/NI to 3.3: Is it likely that missingness in the outcome depended on its true value?**	
**Risk-of-bias judgement**	
Optional: What is the predicted direction of bias due to missing outcome data?	

Domain 4: Risk of bias in measurement of the outcome

Signalling questions	Comments
**4.1 Was the method of measuring the outcome inappropriate?**	
**4.2 Could measurement or ascertainment of the outcome have differed between intervention groups?**	
**4.3 If N/PN/NI to 4.1 and 4.2: Were outcome assessors aware of the intervention received by study participants?**	
**4.4 If Y/PY/NI to 4.3: Could assessment of the outcome have been influenced by knowledge of intervention received?**	
**4.5 If Y/PY/NI to 4.4: Is it likely that assessment of the outcome was influenced by knowledge of intervention received?**	
**Risk-of-bias judgement**	
Optional: What is the predicted direction of bias in measurement of the outcome?	

Domain 5: Risk of bias in selection of the reported result

**Signalling questions**	**Comments**
**5.1 Were the data that produced this result analysed in accordance with a pre-specified analysis plan that was finalized before unblinded outcome data were available for analysis?**	
**Is the numerical result being assessed likely to have been selected, on the basis of the results, from...**	
**5.2. ... multiple eligible outcome measurements (e.g. scales, definitions, time points) within the outcome domain?**	
**5.3 ... multiple eligible analyses of the data?**	
**Risk-of-bias judgement**	
Optional: What is the predicted direction of bias due to selection of the reported result?	

Overall risk of bias

**Risk-of-bias judgement**	
Optional: What is the overall predicted direction of bias for this outcome?	
